# Collaboration to harmonize antimicrobial registry measures (CHARM) database analysis of antibiotic prescribing in urgent and non-urgent care: a retrospective study on demographic factors

**DOI:** 10.1017/ash.2025.10197

**Published:** 2025-11-17

**Authors:** Tan Vo, Kushal Dahal, Michael Klepser, Benjamin Pontefract, Kaylee E. Caniff, Minji Sohn

**Affiliations:** 1 Ferris State University College of Pharmacy, Big Rapids, MI, USA; 2 Collaborative to Advance Pharmacy Enterprises (CAPE), Grand Rapids, MI, USA

## Abstract

**Objective:**

To compare demographic patterns, diagnosis distribution, and prescribing trends between urgent care and non-urgent care clinics for infectious disease encounters across age groups.

**Design:**

Retrospective cross-sectional study.

**Setting:**

Outpatient encounters from 93 facilities (7 urgent care, 86 non-urgent clinics) in a single Michigan health system, from January 2021 to December 2024.

**Participants:**

A total of 161,328 outpatient encounters involving an antibiotic prescription. Data were stratified by age group, sex, race, insurance type, and care setting.

**Methods:**

Antibiotic prescription and ICD-10 diagnosis data were extracted from the CHARM database and compared across settings using χ^2^, Fisher’s exact, and Mann-Whitney tests.

**Results:**

Urgent care encounters involved younger patients (median age 45 vs 55 yr, *p* < .001), with more visits among children aged 0–5 years (9.0% vs 5.3%, *p* < .001). Non-urgent care encounters had more patients aged ≥ 60 years (43.8% vs 34.1%, *p* < .001). Upper respiratory tract infections (URTIs), including acute pharyngitis and otitis media, were more frequent in urgent care (15.0% and 11.9% vs 6.7% and 7.6%, *p* < .001). Urinary tract infections (UTIs) were more common in non-urgent care (15.2% vs 13.8%, *p* < .001). Amoxicillin was the most prescribed antibiotic in urgent care (17.4% vs 11.4%, *p* < .001), while cephalexin led in non-urgent care (13.5% vs 11.5%, *p* < .001).

**Conclusions:**

Comparatively, a larger proportion of urgent care visits were for patients under the age of 18 and for patients with URTIs. Meanwhile, a greater proportion of non-urgent care encounters were for patients over the age of 60 years old and patients with unspecified UTI.

## Introduction

Urgent care clinics have rapidly become a major point of access for acute conditions in the United States, particularly for infectious diseases.^1,2^ Respiratory infections such as influenza-like illnesses, the common cold, and streptococcal pharyngitis are among the most frequent reasons for urgent care visits.^3^ Otitis media (ear infections) and urinary tract infections (UTIs) are also common for urgent care clinics among children and women, respectively.^3^ While non-urgent clinics also treat infectious diseases, patients are often older and managed for chronic conditions.^4^

Understanding how antimicrobial prescribing differs across settings is essential for tailoring antimicrobial stewardship strategies. Previous studies describe differences in patient demographics, visit timing, and access patterns, but there has been limited examination of antibiotic prescribing trends in urgent versus non-urgent care facilities.^5^ This study aims to evaluate demographic trends, infection diagnoses, and antibiotic prescribing patterns across urgent and non-urgent settings within a single healthcare system to identify key areas for antimicrobial stewardship intervention.

## Methods

A retrospective, cross-sectional study was conducted using data from a single health system from January 2021 to December 2024. Data were obtained from the Collaboration to Harmonize Antimicrobial Registry Measures (CHARM) database. The CHARM database extracts antibiotic prescribing data and related information from the electronic medical records (EMRs) of participating outpatient facilities. CHARM supports antimicrobial stewardship by providing participating facilities with timely, standardized dashboards to monitor antibiotic prescribing patterns.^6^ Specific extracted variables included patient age (0–5, 6–17, 18–60, and 60+ years), sex, race, insurance type, day of visit, diagnosis category based on the ICD-10 codes, and prescribed oral antimicrobial. The primary objective was to describe the frequency of infectious disease encounters and antibiotic prescriptions by setting. Comparisons of diagnoses and prescriptions were further stratified by age group to highlight differences.

Descriptive statistics included counts, frequencies, medians, and interquartile ranges (IQR). Group comparisons were conducted using χ^2^ or Fisher’s exact tests (*n* < 5) for categorical variables and Mann-Whitney tests for continuous variables. Analyses were performed in R (R Core Team, 2025) and Microsoft Excel (2021, Professional Plus) with significance defined as *α* < .05. This study received a waiver of consent as it was deemed not human subjects research by the Ferris State University Institutional Review Board.

## Results

**Figure 1.**
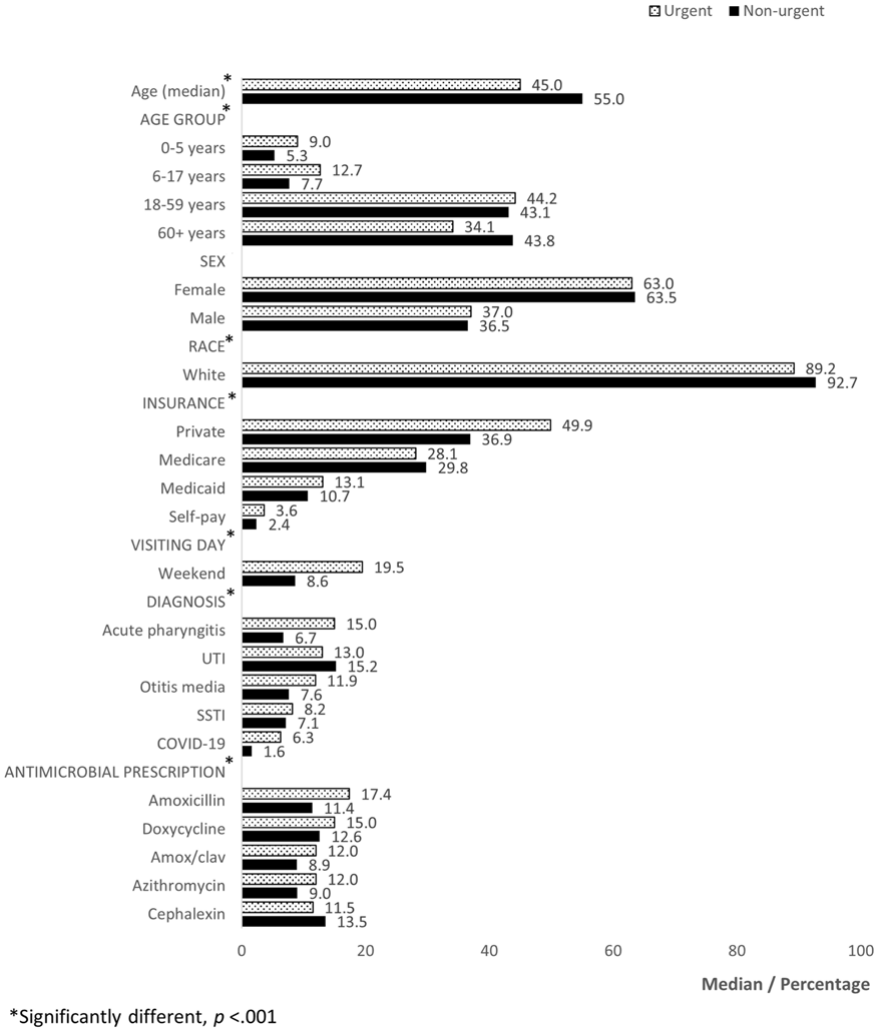
Comparison of urgent and non-urgent encounters, CHARM 2021 – 2024 Bar charts showing differences in patient demographics, diagnosis, and prescription between urgent and non-urgent care settings. Demographic variables include age groups, sex, race, insurance type, and day of visit. Each bar represents the percentage of total encounters within each category. Abbreviations: CHARM, collaboration to harmonize antimicrobial registry measures; UTI, urinary tract infection; SSTI, skin and soft tissue infection; amox/clav, amoxicillin/clavulanate.

Ninety-three facilities were included: 7 urgent care and 86 non-urgent care clinics. There were 161,328 outpatient encounters representing 85,137 patients. Of these, 14.1% occurred in urgent care and 85.9% in non-urgent care. In total, 180,332 antimicrobial prescriptions were issued. See Figure 1 for comparison between settings; more detailed data, including age group stratification, are provided in the Supplemental Appendix. Urgent care patients were younger (median 45 vs 55 yr, *p* < .001), with a greater share of children aged 0–5 (9.0% vs 5.3%, *p* < .001) and 6–17 years (12.7% vs 7.7%, *p* < .001). Patients aged 60 + years accounted for 43.8% of non-urgent encounters compared with 34.1% in urgent care (*p* < .001). No difference was seen in sex distribution between settings; females comprising approximately 63% of all encounters (*p* = .15). Although most visits involved White patients overall, the proportion was lower in urgent care (89.2% vs 92.7%, *p* < .001).

Urgent care encounters were more likely to be associated with private insurance (49.9% vs 36.9%, *p* < .001), Medicaid (13.1% vs 10.7%, *p* < .001), or self-pay (3.6% vs 2.4%, *p* < .001); non-urgent care was more likely associated with Medicare (29.8% vs 28.1%, *p* < .001). Weekend visits were substantially higher in urgent care (19.5% vs 8.6%, *p* < .001).

Infectious disease encounters accounted for most of visits in both care settings. Upper respiratory tract infections (URTIs) such as acute pharyngitis and otitis media were more frequent in urgent care (15.0% vs 6.7%, *p* < .001; 11.9% vs 7.6%, *p* < .001, respectively). COVID-19 was also more common in urgent care (6.3% vs 1.6%, *p* < .001). UTIs were more common in non-urgent care (15.2% vs 13.8%, *p* < .001). Regarding antibiotic prescribing, amoxicillin was most frequently prescribed in urgent care (17.4%), followed by doxycycline (15.0%) and amoxicillin/clavulanate (12.0%). In non-urgent care, cephalexin led (13.5%), followed by doxycycline (12.6%) and amoxicillin (11.4%).

## Discussion

Our study highlights differences between infectious diseases encounters in urgent and non-urgent outpatient care. Urgent care encounters more often involved younger patients, especially children, and were associated with acute respiratory infections. In contrast, non-urgent care served a larger proportion of older adults and was more frequently associated with UTIs. These differences reflect not only patient demographics but also visit timing, as urgent care absorbs more after-hours and weekend demand.^7^ Antibiotic prescribing mirrored these patterns: amoxicillin was more commonly prescribed in urgent care, reflecting its first-line role for pediatric URTIs, whereas cephalexin was more common in non-urgent care, consistent with higher rates of UTIs and skin infections in older populations.^8–10^

This study has several limitations. First, antibiotic appropriateness was not directly linked to specific diagnoses. Additionally, the population consisted of predominantly White patients, limiting generalizability to more diverse regions. Finally, missing or non-specific ICD-10 codes may have led to misclassification of infectious disease encounters. Despite these limitations, the large sample and standardized data provide valuable insights into setting-specific prescribing patterns. Future work addressing these limitations would substantially strengthen these findings by enabling evaluation of concordance with guideline recommendations.

Overall, these findings reinforce the importance of tailoring antimicrobial stewardship interventions by care setting and patient age. Stewardship strategies in urgent care should emphasize judicious prescribing for respiratory conditions, while efforts in non-urgent care may prioritize older adults and urinary or skin infections. Understanding these differences is essential for improving antibiotic use across the continuum of care.^11^

## Supporting information

10.1017/ash.2025.10197.sm001 Vo et al. supplementary material Vo et al. supplementary material

## Supplementary material

To view supplementary material for this article, please visit https://doi.org/10.1017/ash.2025.10197.
