# Supplementary material for: Collaboration to harmonize antimicrobial registry measures (CHARM) database analysis of antibiotic prescribing in urgent and non-urgent care: a retrospective study on demographic factors
Source: Antimicrob Steward Healthc Epidemiol. 2025 Nov 17;5(1):e303. doi: 10.1017/ash.2025.10197 (PMC12645235; doi:10.1017/ash.2025.10197)
Supplement: Vo et al. supplementary material [file S2732494X25101976sup001.docx]

SUPPLEMENTAL APPENDIX

**Supplemental Table 1**. Demographic Characteristics of Study Population with Urgent and Non-urgent Encounters, CHARM 2021-2024

|  | Urgent | Non-urgent | p value |
| --- | --- | --- | --- |
| **Total number of encounters** | 22,748 | 138,580 |  |
| Age (yrs) median (IQR)* | 45 (22-65) | 55 (32-69) | <.001 |
| 0-5 | 2,052 (9.0%) | 7,369 (5.3%) | <.001 |
| 6-17 | 2,880 (12.7%) | 10,690 (7.7%) |  |
| 18-59 | 10,062 (44.2%) | 59,769 (43.1%) |  |
| >60 | 7,754 (34.1%) | 60,752 (43.8%) |  |
| Sex |  |  | 0.15 |
| Female | 14,340 (63.0%) | 88,051 (63.5%) |  |
| Male | 8,408 (37.0%) | 50,529 (36.5%) |  |
| Race |  |  | <.001 |
| White | 20,300 (89.2%) | 128,437 (92.7%) |  |
| Non-White | 250 (1.1%) | 2,205 (1.6%) |  |
| Unknown** | 2,198 (9.7%) | 7,938 (5.7%) |  |
| Insurance |  |  | <.001 |
| Private | 11,359 (49.9%) | 51,182 (36.9%) |  |
| Medicare | 6,397 (28.1%) | 41,285 (29.8%) |  |
| Medicaid | 2,974 (13.1%) | 14,796 (10.7%) |  |
| Self-pay | 826 (3.6%) | 3,280 (2.4%) |  |
| Other/unknown | 1,192 (5.2%) | 28,037 (20.2%) |  |
| Day of arrival |  |  | <.001 |
| Weekday | 18,303 (80.5%) | 126,678 (91.4%) |  |
| Weekend | 4,445 (19.5%) | 11,902 (8.6%) |  |
| Infection-related ICD-10 code | 12,370 (54.4%) | 79,180 (57.1%) | <.001 |
| **Total number of antimicrobial prescriptions** | 24,830 | 155,502 |  |
| Age (yrs) median (IQR) | 45 (22-65) | 55 (32-70) | <.001 |
| 0-5 | 2,220 (8.9%) | 7,960 (5.1%) | <.001 |
| 6-17 | 3,092 (12.5%) | 11,638 (7.5%) |  |
| 18-59 | 11,021 (44.4%) | 67,051 (43.1%) |  |
| >60 | 8,497 (34.2%) | 68,853 (44.3%) |  |
| Sex |  |  | <.01 |
| Female | 15,818 (63.7%) | 100,564 (64.7%) |  |
| Male | 9,012 (36.3%) | 54,938 (35.3%) |  |
| Race |  |  | <.001 |
| White | 22,168 (89.3%) | 144,221 (92.7%) |  |
| Non-White | 269 (1.1%) | 2,480 (1.6%) |  |
| Unknown** | 2,393 (9.6%) | 8,801 (5.7%) |  |
| Insurance |  |  | <.001 |
| Private | 12,349 (49.7%) | 56,543 (36.4%) |  |
| Medicare | 7,024 (28.3%) | 47,092 (30.3%) |  |
| Medicaid | 3,245 (13.1%) | 16,421 (10.6%) |  |
| Self-pay | 905 (3.6%) | 3,632 (2.3%) |  |
| Other/unknown | 1,306 (5.3%) | 31,814 (20.5%) |  |
| Day of arrival |  |  | <.001 |
| Weekday | 19,937 (80.3%) | 142,307 (91.5%) |  |
| Weekend | 4,893 (19.7%) | 13,195 (8.5%) |  |
| Infection-related ICD-10 code | 13,405 (55.0%) | 87,642 (56.4%) | <.001 |
| Abbreviation: IQR: interquartile range *Mann-Whitney U test was used to compare IQR; the rest of categorical variables utilize chi-square test.  **Including unknown/unavailable/other/multiple | | | |

**Supplemental Table 2**. Fifteen Most Common Discharge Antimicrobial Diagnoses Associated with Urgent and Non-urgent Encounters, CHARM 2021-2024

| Diagnosis | Urgent (n = 22,748) | Rank | Non-urgent (n = 138,580) | Rank | p value |
| --- | --- | --- | --- | --- | --- |
| Acute pharyngitis | 3,421 (15.0%) | 1 | 9,227 (6.7%) | 5 | <.001 |
| Urinary tract infection | 3,130 (13.8%) | 2 | 21,071 (15.2%) | 1 | <.001 |
| Unspecified | 1,727 (7.6%) |  | 11,244 (8.1%) |  | <.01 |
| Cystitis | 1,367 (6.0%) |  | 9,265 (6.7%) |  | <.001 |
| Pyelonephritis | 36 (0.16%) |  | 562 (0.41%) |  | <.001 |
| Otitis media | 2,701 (11.9%) | 3 | 10,531 (7.6%) | 2 | <.001 |
| Skin and soft tissue infection | 1,871 (8.2%) | 4 | 9,826 (7.1%) | 3 | <.001 |
| Cellulitis | 1,235 (5.4%) |  | 5,547 (4.0%) |  | <.001 |
| Miscellaneous | 274 (1.2%) |  | 1,719 (1.2%) |  | 0.65 |
| Abscess | 257 (1.1%) |  | 1,777 (1.3%) |  | 0.06 |
| Impetigo, ulcer, MRSA, MSSA, necrotizing fasciitis | 105 (0.46%) |  | 783 (0.57%) |  | 0.05 |
| Genitourinary infection* | 1,934 (8.5%) | 5 | 9,688 (7.0%) | 4 | <.001 |
| Cough | 1,536 (6.8%) | 6 | 5,547 (4.0%) | 6 | <.001 |
| COVID-19 | 1,425 (6.3%) | 7 | 2,242 (1.6%) | 14 | <.001 |
| Acute sinusitis | 1,380 (6.1%) | 8 | 4,539 (3.3%) | 8 | <.001 |
| Upper respiratory tract infection, miscellaneous | 1,355 (6.0%) | 9 | 4,218 (3.0%) | 9 | <.001 |
| Bronchitis | 1,176 (5.2%) | 10 | 3,656 (2.6%) | 10 | <.001 |
| Acute | 732 (3.2%) |  | 1,116 (0.8%) |  | <.001 |
| Unspecified | 442 (1.9%) |  | 2,475 (1.8%) |  | 0.10 |
| Chronic** | 2 (0.01%) |  | 65 (0.05%) |  | <.01 |
| Chronic sinusitis | 1,169 (5.1%) | 11 | 5,484 (4.0%) | 7 | <.001 |
| Dental infection | 640 (2.8%) | 12 | 2,017 (1.5%) | 15 | <.001 |
| Community acquired pneumonia | 449 (2.0%) | 13 | 2,787 (2.0%) | 11 | 0.71 |
| Fever | 396 (1.7%) | 14 | 1,339 (1.0%) | 16 | <.001 |
| Chronic obstructive pulmonary disease, exacerbation | 337 (1.5%) | 15 | 2,254 (1.6%) | 13 | 0.11 |
| *ICD-10 codes involving the genital or urinary tract that were not classified as UTI. This included conditions such as chlamydia, genital herpes, candidiasis, and sex-specific symptoms or infections unrelated to cystitis or pyelonephritis. **Fisher’s exact test; cell value less than 5. | | | | | |

**Supplemental Table 3**. The Five Most Common Antimicrobial Diagnosis by Age Bracket Associated with Urgent and Non-urgent Encounters, CHARM 2021-2024

| Age group  Diagnosis | No. of diagnosis (%) | | p value |
| --- | --- | --- | --- |
|  | Urgent (n=22,748) | Non-urgent (n=138,580) |  |
| 0-5  Otitis media  Acute pharyngitis  URTI, miscellaneous  Cough  Fever | n = 2,052  1,046 (51.0)  527 (25.7)  368 (17.9)  295 (14.4)  158 (7.7) | n = 7,369  4,067 (55.2)  1,186 (16.1)  849 (11.5)  582 (7.9)  494 (6.7) | <.001  <.001  <.001  <.001  .12 |
| 6-17  Acute pharyngitis  Otitis media  Cough  URTI, miscellaneous  Fever*  UTI, unspecified** | n = 2,880  1,268 (44.0)  589 (20.5)  233 (8.1)  200 (6.9)  119 (4.1)  99 (2.9) | n = 10,690  3,611 (33.8)  2,434 (22.8)  603 (5.6)  644 (6.0)  336 (3.1)  390 (3.6) | <.001  <.001  <.001  .07  <.01  .59 |
| 18-59  Acute pharyngitis  Otitis media  Acute sinusitis*  GU, miscellaneous**  UTI, unspecified  UTI, cystitis**  Chronic sinusitis** | n = 10,062  1,321 (13.1)  781 (7.8)  779 (7.7)  751 (7.5)  702 (7.0)  638 (6.3)  657 (6.5) | n = 59,769  3,647 (6.1)  2,962 (5.0)  2,397 (4.0)  2,056 (3.4)  3,922 (6.6)  3,481 (5.8)  2,884 (4.8) | <.001  <.001  <.001  <.001  .12  .04  <.001 |
| >60  COVID-19*  UTI, unspecified  GU, miscellaneous  UTI, cystitis  SSTI, cellulitis  Cough** | n = 7,754  894 (11.5)  887 (11.4)  813 (10.5)  623 (8.0)  555 (7.2)  555 (7.2) | n = 60,752  1,387 (2.3)  6,754 (11.1)  3,929 (6.5)  5,304 (18.7)  2,648 (4.4)  2,435 (4.0) | <.001  .40  .02  .04  <.001  <.001 |
| Abbreviations: URTI: upper respiratory tract infection; GU: genitourinary; UTI: urinary tract infection; SSTI: skin and soft tissue infection  *One of the top five diagnoses in urgent encounters only.  **One of the top five diagnoses in non-urgent encounters only. | | | |

**Supplemental Table 4**. The Ten Most Common Discharge Antimicrobial Prescriptions Associated with Urgent and Non-urgent Encounters, CHARM 2021-2024

| Antimicrobial agent | Urgent (n = 24,830) | Rank | Non-urgent (n = 155,502) | Rank | p value |
| --- | --- | --- | --- | --- | --- |
| Amoxicillin | 4,323 (17.4%) | 1 | 17,759 (11.4%) | 3 | <.001 |
| Doxycycline | 3,716 (15.0%) | 2 | 19,606 (12.6%) | 2 | <.001 |
| Amoxicillin/clavulanate | 2,975 (12.0%) | 3 | 13,830 (8.9%) | 5 | <.001 |
| Azithromycin | 2,973 (12.0%) | 4 | 14,065 (9.0%) | 4 | <.001 |
| Cephalexin | 2,844 (11.5%) | 5 | 20,956 (13.5%) | 1 | <.001 |
| Nirmatrelvir/ritonavir | 1,378 (5.5%) | 6 | 3,233 (2.1%) | 13 | <.001 |
| Nitrofurantoin | 1,260 (5.1%) | 7 | 8,734 (5.6%) | 8 | <.01 |
| Sulfamethoxazole/trimethoprim | 1,201 (4.8%) | 8 | 13,054 (8.4%) | 6 | <.01 |
| Fluconazole | 798 (2.9%) | 9 | 9,779 (6.3%) | 7 | <.001 |
| Cefdinir | 732 (2.9%) | 10 | 3,861 (2.5%) | 12 | <.001 |

**Supplemental Table 5**. The Five Most Common Antimicrobial Prescriptions by Age Bracket Associated with Urgent and Non-urgent Encounters, CHARM 2021-2024

| Age group  Antimicrobial agent | No. of prescription (%) | | p value |
| --- | --- | --- | --- |
|  | Urgent | Non-urgent |  |
| 0-5  Amoxicillin  Cefdinir  Azithromycin  Cephalexin  Amoxicillin/clavulanate | n = 2,220  1,265 (57.0)  277 (12.5)  270 (12.2)  165 (7.4)  110 (5.0) | n = 7,960  4,347 (54.6)  1,230 (15.5)  649 (8.2)  535 (6.7)  613 (7.7) | .05  <.001  <.001  .24  <.001 |
| 6-17  Amoxicillin  Azithromycin  Cephalexin  Amoxicillin/clavulanate  Cefdinir | n = 3,092  1,420 (45.9)  509 (16.5)  370 (12.0)  233 (7.5)  161 (5.2) | n = 11,638  4,675 (40.2)  1,364 (11.7)  1,378 (11.8)  903 (7.8)  990 (8.5) | <.001  <.001  .85  .68  <.001 |
| 18-59  Amoxicillin/clavulanate  Doxycycline  Azithromycin  Amoxicillin*  Cephalexin  Fluconazole** | n = 11,022  1,786 (16.2)  1,626 (14.8)  1,381 (12.5)  1,247 (11.3)  1,109 (10.1)  567 (5.1) | n = 67,051  7,234 (10.8)  9,192 (13.7)  6,066 (9.0)  4,883 (7.3)  7,160 (10.7)  6,479 (9.7) | <.001  <.001  <.001  <.001  <.001  <.001 |
| >60  Doxycycline  Cephalexin  Nirmatrelvir/ritonavir*  Amoxicillin/clavulanate**  Azithromycin  Sulfamethoxazole/trimethoprim**  Nitrofurantoin** | n = 8,496  2,042 (24.0)  1,200 (14.1)  906 (10.7)  846 (10.0)  813 (9.6)  475 (5.6)  497 (5.8) | n = 68,853  9,916 (14.4)  11,883 (17.3)  2,260 (3.3)  5,080 (7.4)  5,986 (8.7)  7,172 (10.4)  5,173 (7.5) | <.001  <.001  <.001  <.001  <.001  <.001  <.001 |
| *One of the top five prescriptions in urgent encounters only.  **One of the top five prescriptions in non-urgent encounters only. | | | |

**Supplemental Figure 1a.** Comparison of urgent and non-urgent encounters by age, CHARM 2021-2024 (urgent encounters)

**

**

**Supplemental Figure 1b.** Comparison of urgent and non-urgent encounters by age, CHARM 2021-2024 (non-urgent encounters)

**

**

**Supplemental Figure 2.** Comparison of most common infectious diagnoses, by care settings and age groups: CHARM 2021-2024

**
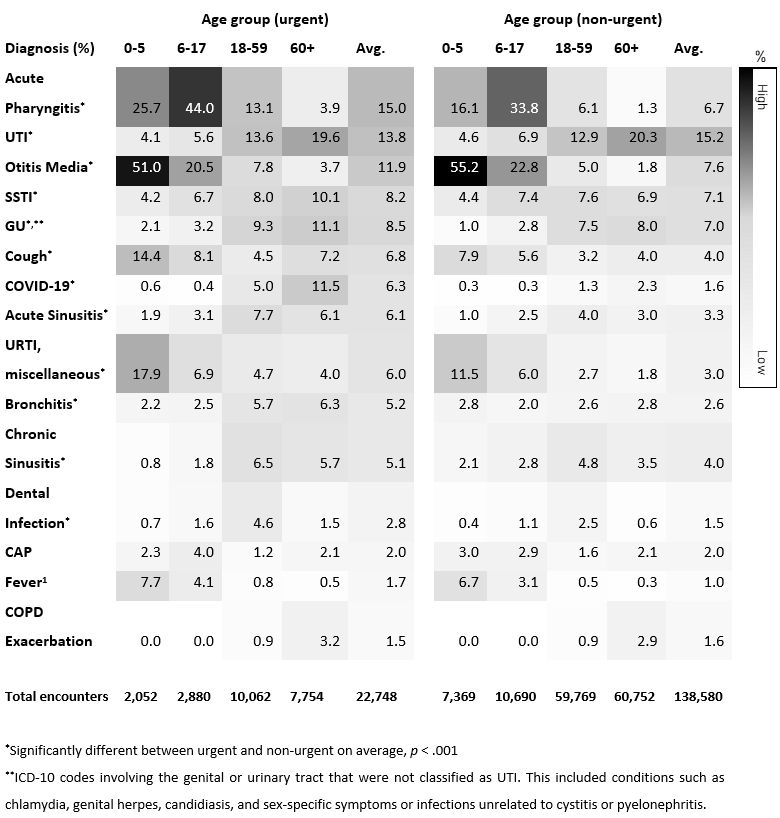
**

**Supplemental Figure 3.** Comparison of most common discharge antimicrobial prescriptions, by care settings and age groups: CHARM 2021-2024

**
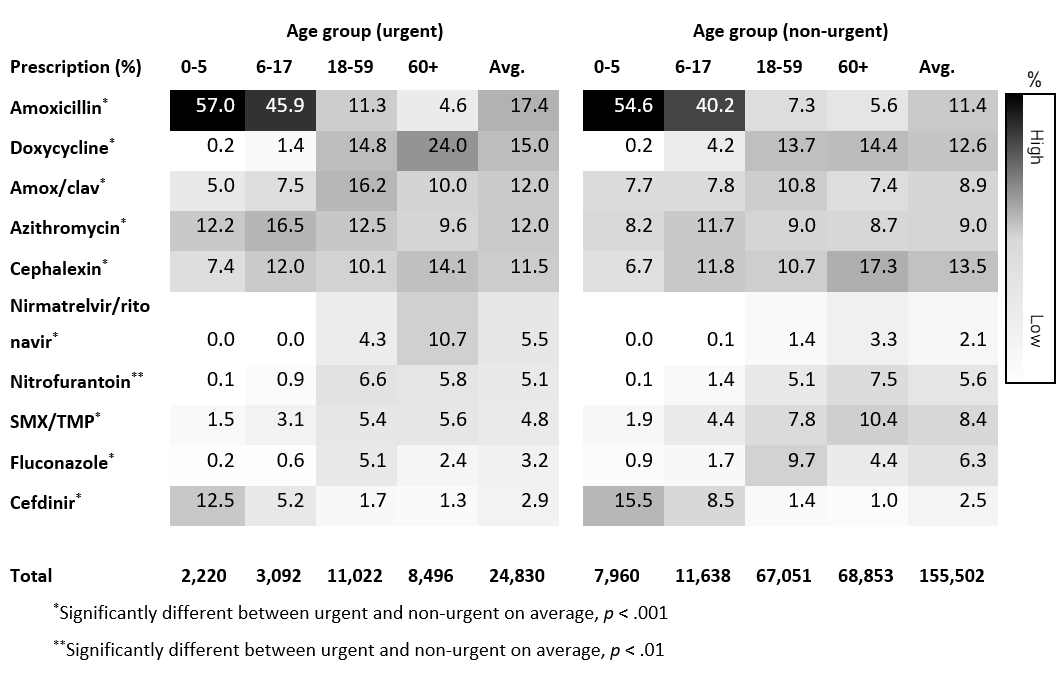
**
